# Hantavirus: biology, immune responses, and pathogenesis

**DOI:** 10.1038/s41419-026-09235-6

**Published:** 2026-09-14

**Authors:** Yufang Shi, Mauro Piacentini, Alimuddin Zumla, Yu Wang, Qing Li, Ying Wang, Jun Zhou, Gerry Melino, Giuseppe Ippolito

**Affiliations:** 1 The Third Affiliated Hospital of Soochow University, Institutes for Translational Medicine, State Key Laboratory of Radiation Medicine and Protection, Biomedical Basic Research Center (BBRC) of Jiangsu, Suzhou Medical College of Soochow University, Suzhou, Jiangsu China; 2 Department of Experimental Medicine and Biochemical Sciences, University of Rome Tor Vergata, Rome, Italy; 3 Institute of Infection, Immunity and Transplantation, University College London, and University College London Hospitals NHS Foundation Trust, London, UK; 4 CAS Key Laboratory of Tissue Microenvironment and Tumor, Shanghai Institute of Nutrition and Health, University of Chinese Academy of Sciences, Chinese Academy of Sciences, Shanghai, China; 5 Unicamillus International Medical University, Rome, Italy

**Keywords:** Viral infection, Immunological disorders

## Abstract

The recent Andes virus (ANDV) outbreak on the MV Hondius cruise has raised global public health alarms due to its dangerous ability to person-to-person spread. Hantavirus infections primarily manifest as hemorrhagic fever with Eurasian renal syndrome (HFRS) and Americas hantavirus cardiopulmonary syndrome (HCPS), both characterized by endothelial manifestation. Two principal mechanisms, virus-mediated cytopathy and host-driven immunopathology, have been proposed to account for the pathological progression, while their interplay is yet to be fully explored. In this review, we synthesize the recent research on hantavirus biology, cellular entry mechanisms, and epidemiological characteristics, and particularly highlight that viral immune evasion and subsequent uncontrolled host immune responses act as the core pathogenic drivers. Hantaviruses employ diverse strategies to antagonize type I interferon signaling and autophagy, which delay early innate immune activation and enable extensive viral dissemination. The excessive activation of cytotoxic CD8⁺ T cells, natural killer (NK) cells, combined with functionally impaired regulatory T cells and dysregulated humoral immunity, initiates severe inflammatory cascades, which in turn disrupt endothelial barrier integrity and cause organ vasculopathy. We also outline current therapeutic and preventive strategies, along with key directions for future research. A better understanding of hantavirus immunopathogenesis will help develop improved countermeasures against this zoonotic disease.

## Facts

- **Paradox of Hantavirus Pathogenesis:** Based on current epidemiological and clinical evidence, this review highlights the central paradox of host–pathogen interactions, wherein hantaviruses cause severe immunopathology in humans while maintaining asymptomatic persistent infections in rodent reservoirs.
- **Shift to an Immunopathogenic Framework:** To better elucidate the mechanisms of vascular breakdown, this review proposes a transition from a virus-centric model to an immune-mediated framework, systematically integrating early viral IFN-I evasion, excessive CD8⁺ T cell/NK cell activation, and Treg deficiency.
- **Therapeutic Potential and Research Priorities:** While targeted immunomodulators and novel single-dose mRNA vaccines show promise in mitigating severe disease, precise mapping of the receptor-tropism-pathogenesis axis, immune calibration kinetics, and host tolerance mechanisms remains critical for future clinical interventions.

## Introduction: the central paradox of hantavirus pathogenesis

Hantaviruses present a classic paradigm in emerging infectious disease biology, where the central mechanistic puzzle remains fundamentally unresolved: how does a pathogen that establishes persistent, asymptomatic infection in its natural rodent reservoirs trigger severe, frequently lethal immunopathology upon spillover into accidental human hosts [1]? This biological dichotomy sits at the critical intersection of viral molecular biology, host-endothelial dynamics, and immunopathogenesis—domains that have generated competing explanatory frameworks over four decades of research [1, 2].

The two clinically distinct manifestations—hemorrhagic fever with renal syndrome (HFRS) in Eurasia and hantavirus cardiopulmonary/pulmonary syndrome (HCPS/HPS) in the Americas—share an underlying mechanism of endothelial barrier disruption, yet diverge in target organ tropism and severity profiles [1, 2].

The field has oscillated between two dominant explanatory paradigms: a virus-centric model emphasizing direct cytopathic effects and a host-centric model foregrounding immune-mediated vascular damage. Contemporary evidence increasingly favors a synthesis in which viral immune evasion strategies paradoxically amplify subsequent immunopathology, but the precise mechanistic architecture of this interplay remains contested [3–5].

The urgency of resolving these contested mechanistic details has been underscored by an atypical, high-profile outbreak in May 2026 aboard the cruise ship MV *Hondius* [6]. This cluster, rapidly traced to Andes virus (ANDV) infection, has renewed focus on the unique biological features of New World hantaviruses—particularly their capacity for person-to-person transmission within closed environments [6, 7].

Here, we synthesize the current scientific literature to map the evolving research landscape on hantaviruses across six key interconnected domains: viral biology and molecular structure, mechanisms of cellular entry and infection, innate and adaptive immune responses, pathogenic mechanisms of vascular leakage, global epidemiological trends, and emerging therapeutic and prophylactic strategies.

## Viral biology: molecular architecture and evolutionary constraints

### Genomic organization and structural determinants

**Fig. 1:**
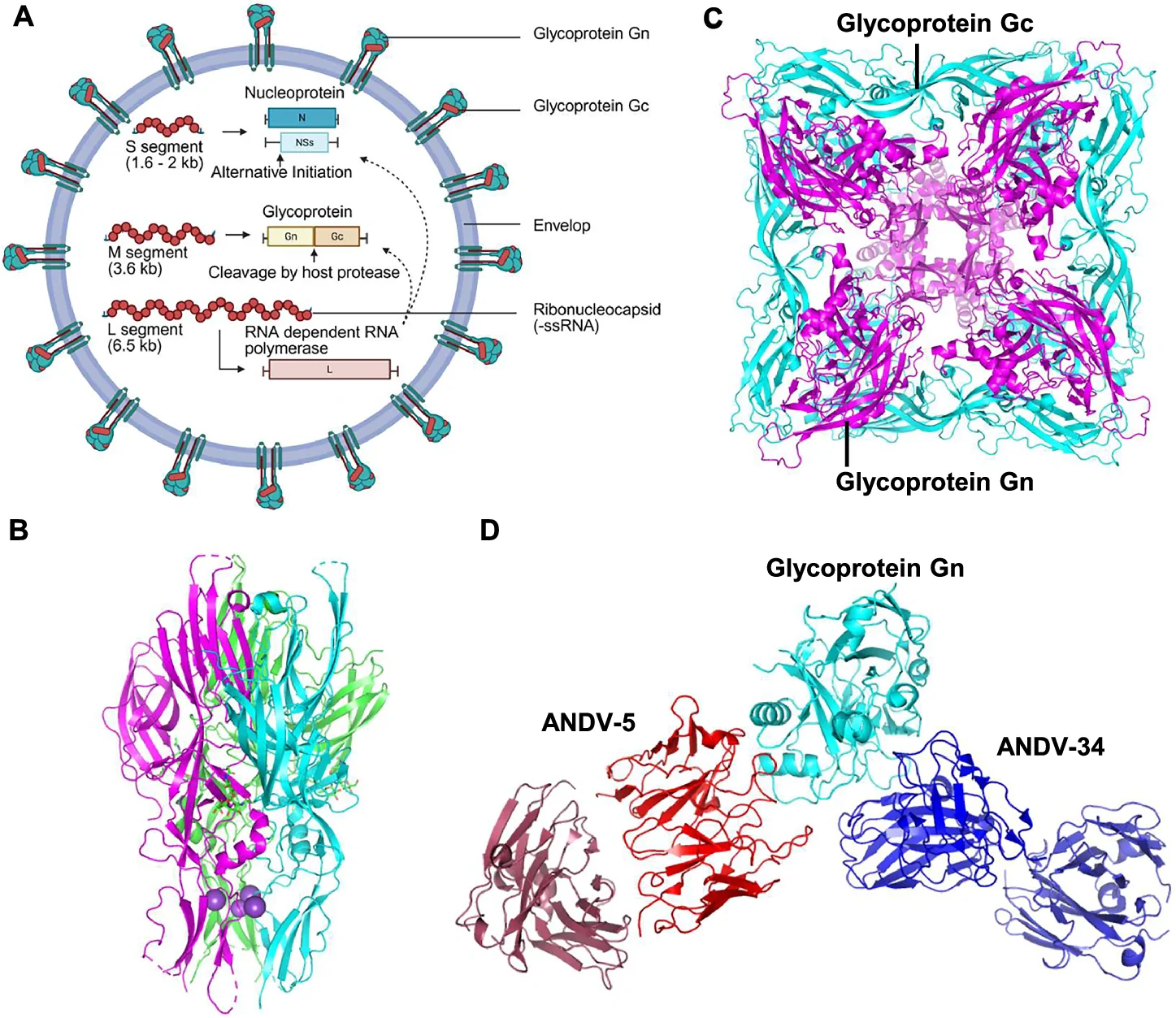
Structural characteristics of hantavirus and its key proteins. **A** Schematic representation of the Orthohantavirus Andesense virion structure and genome organization. (Adapted and modified from https://viralzone.expasy.org/11847). **B** Structure of hantavirus envelope glycoprotein Gc in postfusion conformation in presence of 600 mM KCL (PDB: 5LJX). **C** High-resolution in situ ANDV single tetramer structure (PDB: 9P3I). **D** CryoEM structure of Hantavirus ANDV Gn(H) protein complex with 2Fabs ANDV-5 and ANDV-34 (PDB: 8DBZ).

Hantaviruses are enveloped negative-sense single-stranded RNA viruses within the *Hantaviridae* family of the order *Bunyavirales* [4, 8, 9]. The genome contains three segments: the large (L) segment for RNA-dependent RNA polymerase (RdRp), the medium (M) segment for glycoprotein precursor (GPC, cleaved co-translationally into envelope glycoproteins Gn and Gc), and the small (S) segment for nucleocapsid protein (NP)(Fig.1A). This tripartite segmented architecture permits genetic reassortment, conferring evolutionary flexibility that may generate viral variants with novel pathogenic properties [10].

The glycoproteins Gn and Gc are critical determinants of host cell tropism and are the primary targets for neutralizing antibody responses (Figs. 1B – D) [11, 12]. The M segment thus occupies a central position in pathogenicity determination: it encodes the molecules responsible for cellular attachment, membrane fusion, and the principal neutralization epitopes. Point mutations in glycoprotein sequences can profoundly alter viral infectivity, as demonstrated by studies showing that two amino acid substitutions in Old World hantavirus glycoproteins are sufficient to generate highly infectious recombinant vectors [13].

The evolutionary stability of these segments is illustrated by real-time genomic sequencing from the May 2026 MV *Hondius* outbreak cluster. Genomic sequencing and characterisation complemented the epidemiological investigation into the point of origin and subsequent transmission [14]. Each of the S, M, and L genomic segments from the three hantavirus isolates displays high sequence consistency. This observation suggests that the virus has not undergone reassortment, an evolutionary event in which segmented viral strains swap genetic fragments to generate new variants. Accordingly, the virus linked to this outbreak most probably originates from a single genetically stable lineage, instead of arising from recent genetic recombination between distinct hantavirus strains [14].

### The RdRp and cap-snatching mechanism

The L segment-encoded RdRp mediates both transcription and replication. A distinctive feature of hantavirus mRNA synthesis is the cap-snatching mechanism, whereby the viral RdRp cleaves short capped RNA primers from mRNAs of host origin to initiate viral transcription. This process requires host cell factors, establishing a fundamental dependency on the cellular transcriptional machinery [15]. This host factor requirement for cap snatching represents a potential therapeutic vulnerability, as disruption of this interaction could selectively inhibit viral replication without directly targeting host processes.

### Host reservoir biology and spillover dynamics

Each hantavirus species has co-evolved with a specific rodent host reservoir in which persistent, asymptomatic infection is maintained (Table 1) [16]. The striped field mouse (*Apodemus agrarius*) serves as the main natural reservoir host of Hantaan virus (HTNV). In European regions, bank voles (*Myodes glareolus*) act as persistent carriers that preserve Puumala virus (PUUV) [16].

**Table 1:** Evolutionary distribution, geographic reservoir segregation, and target organ disease phenotypes of major pathogenic hantaviruses.

Viral Species Lineage	Primary Natural Reservoir Host	Vector Family Taxonomy	Geographic Distribution	Primary Human Clinical Manifestation	Target Organ Vulnerability
Hantaan virus (HTNV)	Apodemus agrarius (Striped field mouse)	Muridae	Eurasia	Hemorrhagic Fever with Renal Syndrome (HFRS)	Renal Glomerular Microvasculature
Puumala virus (PUUV)	Myodes glareolus (Bank vole)	Cricetidae	Europe	Nephropathia Epidemica (Mild HFRS)	Renal Tubules & Epithelial Junctions
Sin Nombre virus (SNV)	Peromyscus maniculatus (Deer mouse)	Cricetidae	North America	Hantavirus Pulmonary Syndrome (HPS/HCPS)	Pulmonary Endothelium & Alveoli
Andes virus (ANDV)	Oligoryzomys longicaudatus (Long-tailed colilargo)	Cricetidae	South America	Hantavirus Pulmonary Syndrome (HPS/HCPS)	Systemic Vasculature & Airway Monolayers

Long-term co-speciation within distinct small mammal families ensures persistent, non-lytic asymptomatic carriage in specific hosts. Accidental exposure of the human vascular network to aerosolized excreta from these specific reservoirs determines whether pathology shifts toward Eurasia-centric renal damage or Americas-centric cardiopulmonary syndrome.

The ANDV virus is carried by *Oligoryzomys longicaudatus*, a wild rodent commonly called the long-tailed colilargo. The geographical range of this species extends from northern Chile and northwestern Argentina where there is a stable population. This rodent is not naturally present in Europe and in the United States, and established wild populations of this species are completely absent abroad. This is crucial since introduction to local rodent populations and potential subsequent rodent-to-human transmission in Europe is not expected [16].

Human infection is accidental, occurring primarily through inhalation of aerosolized virus-containing rodent excreta (urine, feces, saliva) [4, 16]. The mechanisms by which reservoir hosts suppress pathology while maintaining viremia—likely involving regulatory T cell-mediated immunosuppression—stand in stark contrast to the hyperinflammatory response in humans [3, 11]. Understanding this dichotomy remains one of the field’s central theoretical challenges.

Sustained human-to-human transmission has been validated only for ANDV among hantavirus species [7, 17]. The outbreak on the MV *Hondius* is considered a case study of a potential superspreader scenario, allowing the effects of prolonged case contact, closed ventilation, delayed symptom appearance and late diagnosis to be studied in a real-life context. All conditions were present to allow secondary cases aboard the ship and tertiary cases across international borders [7].

## Mechanisms of cellular entry and tropism

### Receptor utilization and endothelial tropism

**Fig. 2:**
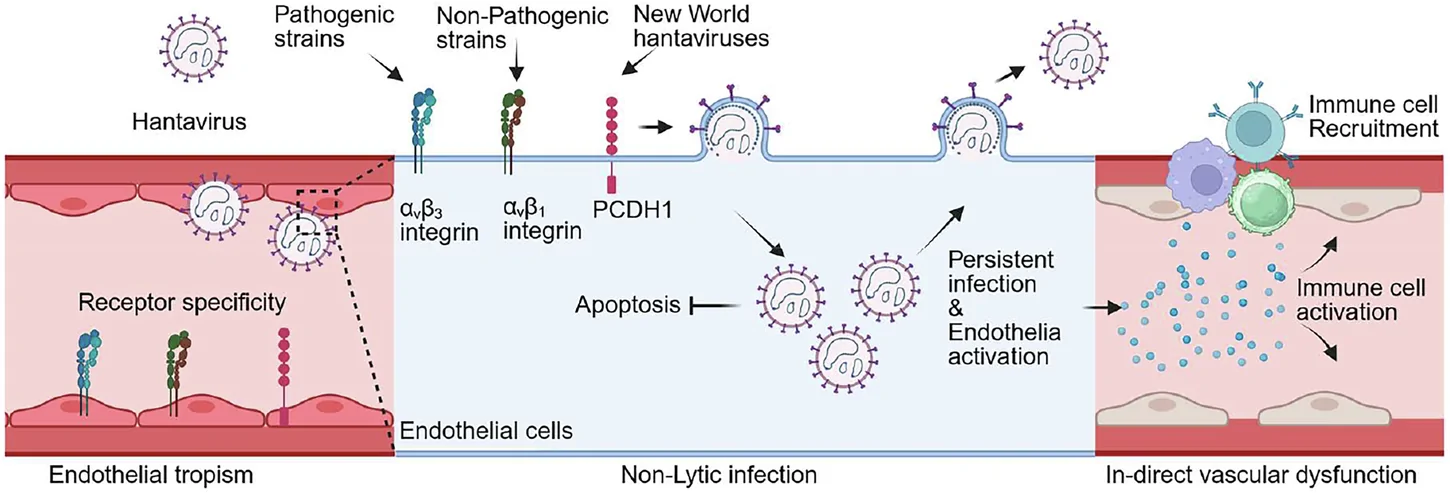
Mechanisms of cellular entry and tropism. Schematic depicting hantavirus infection of endothelial cells. Receptor specificity (α_v_β_3_, α_v_β_1_, and PCDH1) drives endothelial tropism. Infection is non-lytic, with viral inhibition of apoptosis promoting persistence. Pathogenesis arises from indirect, immune-mediated endothelial activation and vascular dysfunction, rather than direct cytopathology. (Created in https://BioRender.com).

Hantaviruses preferentially target endothelial cells, which constitutes the mechanistic basis for the vascular pathology that defines both HFRS and HCPS [4]. The primary cellular receptors for pathogenic hantaviruses include α_v_β_3_ integrin for pathogenic strains, and α_v_β_1_ integrin for non-pathogenic strains, and latest work identifies PCDH1 as a key receptor governing the cellular entry of New World hantaviruses [18]. This receptor specificity provides a molecular explanation for endothelial tropism and suggests that the tropism itself—rather than any direct cytopathic mechanism—positions the virus to trigger vascular dysfunction through immune-mediated collateral damage (Fig. 2).

### Non-lytic infection and its pathogenic implications

A critical distinguishing feature of hantavirus biology is that infection of endothelial cells is non-cytopathic [19]. Unlike many hemorrhagic fever viruses that destroy target cells directly, hantaviruses establish productive infection without inducing overt cellular damage. This non-lytic character has profound implications for pathogenesis theory: it necessarily redirects pathogenic mechanisms toward indirect, immune-mediated injury rather than direct viral cytopathology (Fig. 2). Furthermore, hantaviruses actively inhibit apoptosis in infected cells, promoting viral persistence and potentially amplifying subsequent immune-mediated damage when cytotoxic responses eventually target infected endothelium [20].

## Immune responses: the double-edged sword

**Fig. 3:**
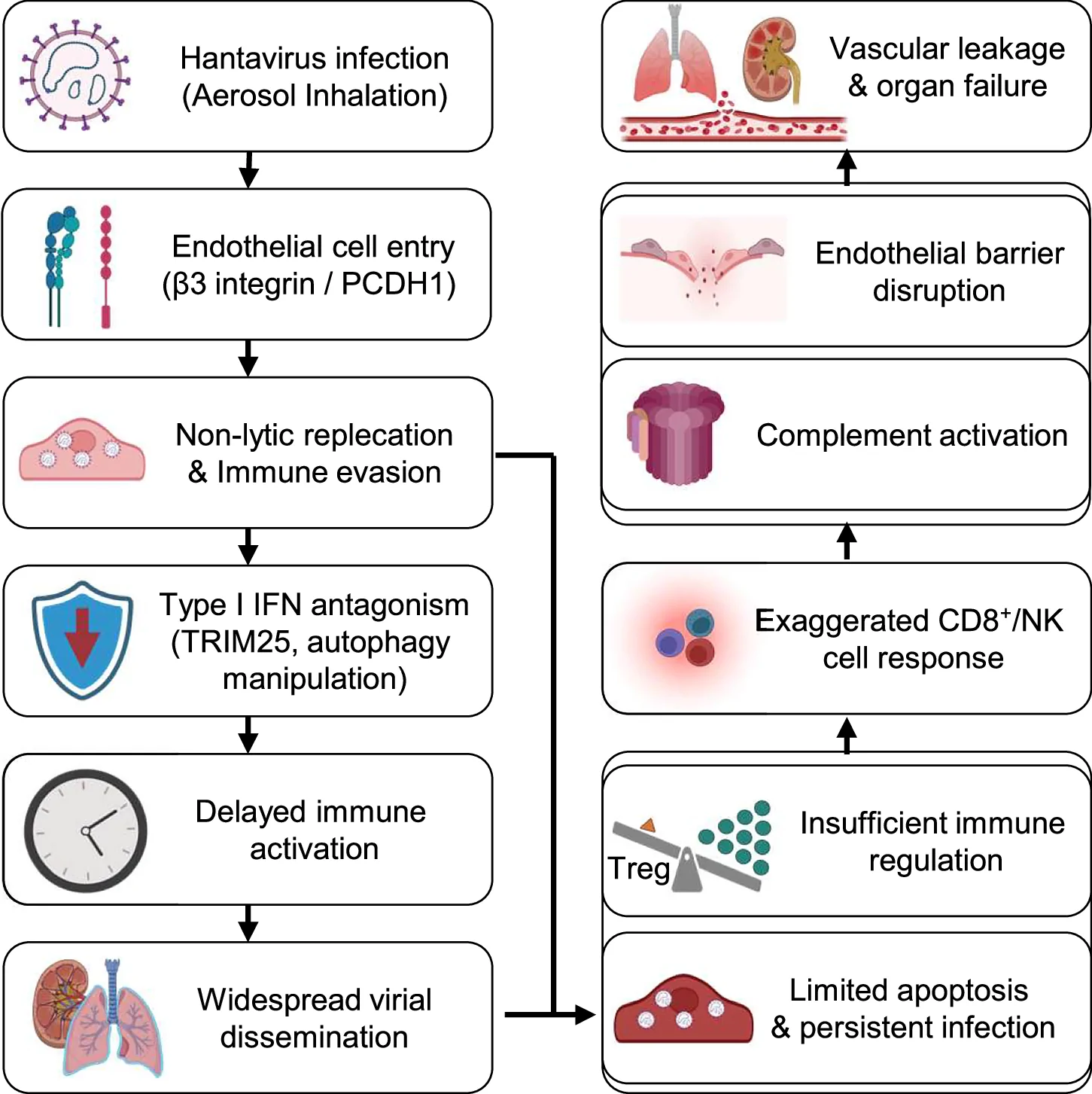
Immune evasion and immunopathogenesis of hantavirus infection. Hantaviruses enter endothelial cells and replicate with limited cytopathic effect. Early antagonism of type I interferon responses may delay innate immune activation and facilitate viral dissemination. Persistent infection, insufficient immune regulation, exaggerated CD8⁺/NK cell responses, and complement activation converge to disrupt endothelial barrier integrity, leading to vascular leakage and organ failure. (Created in https://BioRender.com).

Hantavirus immunopathogenesis is driven by viral immune evasion and subsequent host immune dysregulation. Suppression of type I interferon signaling by the virus promotes systemic dissemination. Together with regulatory T cell deficiency and hyperactivation of CD8⁺ T cells, NK cells, and B cells, these cells elicit exaggerated cytotoxic responses, which ultimately cause endothelial damage, complement activation and vascular leakage in severe infections (Fig. 3).

### Innate immune activation and viral evasion

Hantaviruses have evolved sophisticated mechanisms to evade and delay innate immune detection, particularly the type I interferon (IFN-I) response [21]. The IFN-I signaling pathway, mediated through RIG-I-like receptors (RLRs), is the primary cellular sensor for hantavirus RNA. However, multiple viral proteins actively antagonize this pathway: the glycoprotein cytoplasmic tail inhibits TRAF3/TBK1-directed IRF3 activation, while the nucleocapsid protein (NP) sequesters double-stranded RNA intermediates to prevent RLR activation [22].

Recent work has identified TRIM25 as a specific target of Hantaan virus immune evasion. HTNV inhibits the type I interferon response by targeting RLR signaling pathways through TRIM25 [21]. Furthermore, the glycoprotein and nucleocapsid protein encoded by hantaviruses modulate autophagic flux to suppress the host’s innate immune reactions [23]. By disrupting normal autophagic signaling, these viral proteins suppress IFN-I production and inflammatory cytokine secretion, creating a permissive environment for early viral replication.

The theoretical significance of these evasion strategies extends beyond mere immune escape. By delaying the initial innate response, hantaviruses allow greater viral dissemination before adaptive immunity is engaged. When the adaptive response finally mounts, it confronts a more widely distributed infection, necessitating a more vigorous effector response—one that, in the context of endothelial tropism, inevitably causes collateral vascular damage. This “delay-and-amplify” model represents the current leading framework for understanding hantavirus immunopathogenesis [3, 5].

### Mononuclear phagocyte redistribution

A marked redistribution of peripheral mononuclear phagocytes (MNPs) to the airways occurs during acute Puumala virus (PUUV) infection, which likely drives localized immune activation at the initial infection focus [3]. Pathogenic hantaviruses can replicate within cells of the mononuclear phagocyte system (MPS), and this replication capacity has been proposed as a determinant of pathogenicity [24]. The ability to productively infect and disseminate through MPS cells may explain systemic viral spread and the multi-organ involvement characteristic of severe hantavirus disease.

### Cytotoxic lymphocyte responses: CD8^+^ T cells and NK cells

The adaptive immune response to hantavirus infection is dominated by a vigorous cytotoxic lymphocyte response involving both CD8^+^ T and NK cells. Bronchoalveolar lavage (BAL) studies in patients with acute PUUV infection have revealed increased numbers of activated CD8^+^ T and NK cells, along with elevated granzyme A and granzyme B [25]. Critically, the level of this cytotoxic lymphocyte response in pulmonary correlates directly with disease severity and systemic organ dysfunction.

The observed link between the magnitude of host immune activation and disease severity represents compelling evidence in favor of the immunopathogenic mechanism. RNA of PUUV was detectable in BAL cellular specimens, with viral loads showing a negative correlation with T-cell-mediated immune responses—suggesting that while effector T cells control viremia, they simultaneously drive tissue damage. NK cell activation is excessive and persistent, with highly elevated numbers persisting in peripheral blood following PUUV infection [3].

### Regulatory T cell deficiency

Notably, patients with hantavirus infection exhibit reduced regulatory T cell (Treg) relative to healthy individuals. This deficiency in immune regulation may reflect an inadequate counter-regulatory response during acute infection, permitting the unchecked expansion of cytotoxic effector populations. The contrast with rodent reservoir hosts—in which regulatory mechanisms suppress immunopathology despite persistent viremia—highlights Treg dynamics as a potential mechanistic fulcrum distinguishing protective from pathological responses [26, 27].

### Humoral immunity and B cell responses

Hantavirus infection elicits a rapid virus-specific humoral response characterized by early IgM production and subsequent IgG seroconversion during the convalescent phase. Additionally, the expansion of plasma cells and CD27⁻IgD⁻ B cell populations following hantavirus exposure correlates with unique pathological alterations in patients, particularly thrombocytopenia and transient kidney dysfunction [28]. Neutralizing antibodies primarily target the Gn and Gc glycoproteins and are considered protective, as evidenced by the therapeutic efficacy of passive antibody transfer in animal models [12]. While neutralizing antibodies contribute to viral clearance, the broader B cell activation and immune complex formation may contribute to complement activation and endothelial damage [29].

## Pathogenesis: endothelial dysfunction as the unifying mechanism

### The endothelial barrier disruption paradigm

The pathological hallmark shared by both HFRS and HCPS is increased vascular permeability leading to plasma leakage, edema, and hypovolemic shock [1, 2]. The critical insight—now supported by multiple lines of evidence—is that this vascular leakage occurs in the absence of overt endothelial cell death [19]. Instead, the endothelial barrier is functionally disrupted through alterations in intercellular junction integrity, glycocalyx degradation, and cytokine-mediated permeability changes [19]. Suppression of thrombospondin-1 (THBS1) occurs in a strain-specific manner during hantavirus infection of cultured endothelial cells, which directly interferes with blood homeostasis cascades and may amplify coagulation abnormalities without cell death.

### The immunopathogenic model: synthesis of evidence

**Fig. 4:**
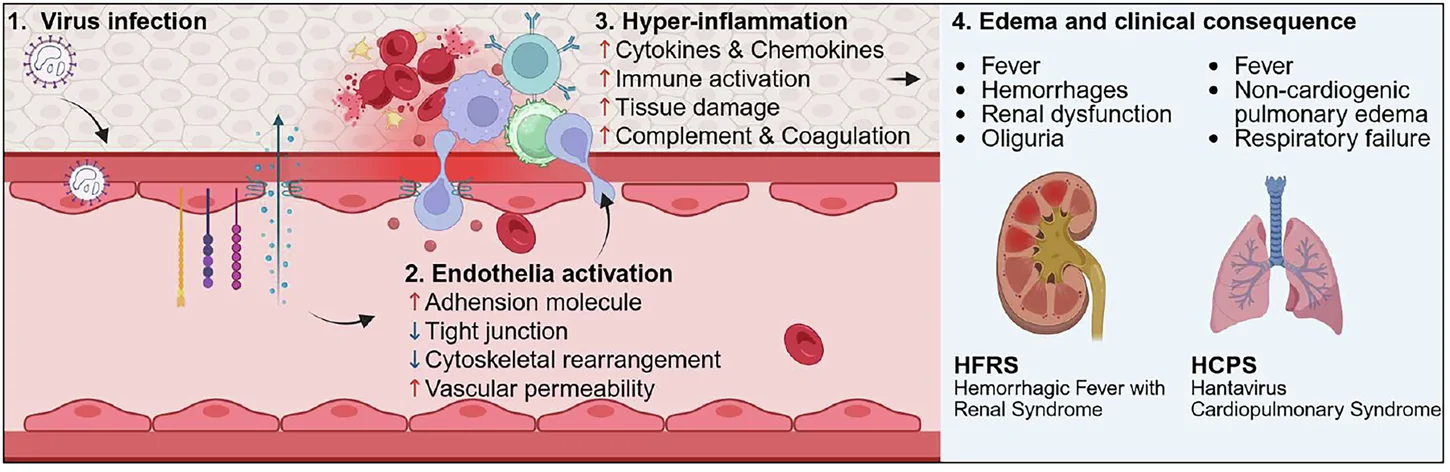
Progressive steps in hantavirus pathogenesis. The schematic illustrates the cascade of events leading to HFRS or HCPS [1]. Hantavirus infects endothelial cells [2]. Infection induces endothelial activation [3]. Hyper-inflammation amplifies vascular injury, increasing vascular permeability [4]. End-organ edema results in HFRS (renal involvement) or HCPS (pulmonary failure). (Created in https://BioRender.com).

The contemporary model of hantavirus pathogenesis integrates viral and host factors into a multi-step cascade (Fig. 4):

- Initial immune evasion: Viral proteins suppress IFN-I responses, allowing viral dissemination through endothelium and MPS cells.
- Delayed but excessive immune activation: When innate and adaptive responses engage, they encounter widespread endothelial infection, triggering massive cytotoxic lymphocyte recruitment to infected vascular beds.
- Effector-mediated barrier disruption: CD8^+^ T cells and NK cells release granzymes and pro-inflammatory cytokines TNFα, IFNγ that disrupt endothelial junctions while targeting infected cells.
- Amplification through complement and coagulation: Complement activation, platelet consumption, and coagulopathy amplify vascular damage.
- Inadequate immune regulation: Treg deficiency permits unchecked expansion of effector responses.

This framework explains why disease severity peaks after viral load begins declining—the immunopathological phase is temporally dissociated from peak viremia [4].

### Organ-specific pathology: HFRS versus HCPS

The divergence between HFRS (primarily renal involvement) and HCPS (primarily pulmonary involvement) likely reflects differences in glycoprotein-mediated tissue tropism encoded by the M segment. In HFRS, the major clinical manifestations include acute kidney injury, coagulation abnormalities, and variable degrees of hemorrhage, with a case fatality rate of 5–10% depending on the causative viral species [1, 2]. In HCPS, rapid onset non-cardiogenic pulmonary edema dominates, with fatality rates reaching 35–40% [1, 2]. The pathophysiology of hantavirus-induced kidney disease represents a continuum from subclinical urinary abnormalities to severe acute kidney injury, with up to 60% of renal damage manifesting as pathological urinary sediment changes without meeting criteria for Acute Kidney Injury (AKI).

### Genetic susceptibility

Human leukocyte antigen (HLA) haplotypes is also reported to be associated with disease severity, suggesting that the magnitude and quality of adaptive immune responses—shaped by antigen presentation capacity—modulate pathogenic outcomes [30]. This genetic dimension further supports the immunopathogenic model: if disease were purely virus-driven, HLA polymorphisms would be less relevant. Certain HLA alleles may promote more efficient presentation of hantavirus epitopes, leading to more vigorous (and more damaging) T cell responses.

## Epidemiology: global distribution and outbreak dynamics

### Geographic distribution and disease burden

Hantaviruses cause approximately 200,000 human infections worldwide annually [8]. HFRS predominates in Asia (particularly China, which reports the vast majority of global cases) and Europe, while HCPS occurs exclusively in the Americas. Eight countries in the Americas recorded 229 confirmed hantavirus cases and 59 deaths in 2025, with a case fatality rate of 25.76%. Europe registered 1,885 cases in 2023, corresponding to an incidence of 0.4 cases per 100,000 people, which was the lowest rate between 2019 and 2023. Hemorrhagic fever with renal syndrome (HFRS) is primarily caused by Dobrava-Belgrade virus (DOBV), Hantaan virus (HTNV), Puumala virus (PUUV), and Seoul virus (SEOV), while Andes virus (ANDV) and Sin Nombre virus (SNV) are the major causative pathogens of hantavirus cardiopulmonary syndrome (HCPS) [6].

### Ecological drivers of outbreaks

Hantavirus outbreaks are fundamentally linked to rodent population dynamics, which in turn are driven by climatic and ecological factors [31]. Mast seeding events, favorable climate conditions, and land-use changes that increase human-rodent contact all modulate outbreak risk. The concept of “spillover events”—whereby ecological perturbations drive increased rodent populations and contact with humans—frames hantavirus epidemiology within a broader One Health context.

### Person-to-person transmission (PTPT)

A distinctive epidemiological feature is that most hantaviruses are not transmitted between humans. However, Andes virus (ANDV) in South America represents a notable exception, with documented human-to-human transmission that has implications for outbreak management and pandemic preparedness. The most recent spread of ANDV on the MV *Hondius* ship is clearly the case (Table 2). Studies published on the PTPT of the Andes virus in Argentina are critical for establishing effective public health interventions, providing the genomic, epidemiological and clinical data necessary to track infection chains and design containment strategies during outbreaks [7, 17].

**Table 2:** Epidemiological profile of the MV *Hondius* transmission chain.

Outbreak Parameter	Field Record Metrics & Data Points	Epidemiological Assessment & Biosecurity Context
Primary Etiology	Andes virus (ANDV); single stable lineage.	Full-genome sequencing confirmed identical S, M, and L configurations across patients, ruling out active reassortment.
Cluster Size	13 human cases (12 laboratory-confirmed via RT-PCR, 1 classified as probable).	Highlights how effectively New World hantaviruses can spread within confined environments.
Clinical Profile	Fever, acute gastrointestinal distress, rapid progression to ARDS, hypovolemic shock, and severe pneumonia.	Reflects the high virulence and severe clinical kinetics characteristic of New World cardiopulmonary lineages.
Point of Spillover	Landfill site tracking in Ushuaia, Argentina (pre-boarding bird-watching tour).	Confirms original exposure occurred via rodent vectors before embarkation, matching the known incubation period.
Secondary Vector Path	Confirmed person-to-person transmission (PTPT) in closed environments.	Supports calls to re-evaluate public health transmission models and implement strict airborne infection controls at sea.
Mortality Analysis	3 total fatalities (2 PCR-confirmed, 1 probable case); 23% Case Fatality Ratio (CFR).	Underscores the public health risks associated with New World hantaviruses and current gaps in outbreak preparedness.

Profile of the May 2026 maritime outbreak cluster highlighting the risks of secondary person-to-person transmission. This event serves as a critical case study for investigating transmission heterogeneity and risk management in closed common ventilation environments.

Recent systematic reviews analyzed studies from Argentina and Chile to establish clear parameters for interpersonal contact and transmission [17]. One cause for concern for PTPT is that recent studies on ANDV genetic mutations have demonstrated, using animal models, that the virus can undergo attenuation traceable to specific mutations in the genome [32]. Such adaptive virulence mutations could predict increased transmissibility in humans [32].

## Therapeutic strategies: current landscape and emerging approaches

### The therapeutic vacuum

Despite decades of research, no FDA-approved specific antiviral therapy or vaccine exists for hantavirus disease [9, 33]. This therapeutic vacuum reflects both the relatively low case numbers (limiting commercial incentive) and the complexity of pathogenesis (where antiviral and immunomodulatory approaches must be balanced). Treatment remains primarily supportive, centered on careful fluid management and organ support [8, 9]. Respiratory support, ranging from non-invasive oxygenation to advanced mechanical ventilation or Extracorporeal Membrane Oxygenation (ECMO), is vital to mitigate acute respiratory distress in HCPS [9]. In parallel, clinical management of HFRS demands strict fluid and electrolyte regulation, supplemented by renal replacement therapy or hemodialysis to counteract fluid overload and uremia during acute renal dysfunction [9]. Beyond these critical supportive measures, targeted antiviral strategies, novel immunotherapeutic approaches, and vaccine candidates are actively being developed to address the therapeutic vacuum.

### Antiviral approaches

- **Ribavirin** has shown efficacy in reducing mortality from HFRS in some clinical studies but has proven largely ineffective against HCPS [9]. This differential efficacy may relate to the temporal dynamics of pathogenesis: in HCPS, the life-threatening phase is driven by immunopathology that peaks after viral load has already begun declining, rendering antivirals ineffective at the critical disease stage.
- **Favipiravir**, a drug approved in Japan to treat influenza, showed real promise in blocking Andes virus [34]. As a wide-spectrum antiviral agent, favipiravir targets RNA-dependent RNA polymerase to halt viral replication processes. This drug has shown in vitro efficacy against many RNA viruses and is from time to time proposed for high-consequence viral infections, from Lassa to Rift Valley Fever virus.
- **Urolithin B**, a phenolic compound produced in the human gut, showed active promise in blocking Andes virus, inhibiting the infection and restoring normal metabolism in infected cells while leaving healthy cells largely unchanged [35].
- **Morin**, a plant-derived natural flavonoid, suppresses Hantaan virus (HTNV) replication at the post-entry phase of its life cycle. It binds directly to the HTNV nucleocapsid protein (NP) to execute antiviral activity, making it a promising candidate for subsequent therapeutic research [36].

### Immunotherapeutic approaches

Given the dominant role of immunopathology in disease severity, immunomodulatory strategies have gained theoretical prominence. The observation of similarities between severe hantavirus infections and other hyperinflammatory syndromes (such as macrophage activation syndrome and cytokine storm in sepsis) suggests that therapeutic interventions successful in those conditions—such as IL-6 receptor blockade, JAK inhibitors, or corticosteroids—might be applicable [3]. Passive immunotherapy using neutralizing monoclonal antibodies represents perhaps the most advanced approach, showing protective efficacy in animal models [9, 12].

### Vaccine development

The development of vaccines has been limited due to technical and stringency problems in virus isolation, large-scale cell culture, biosecurity requirements, and the difficulty in performing clinical trials. Three different technologies—inactivated vaccines, viral vector-based vaccines, and subunit platforms—have been used for years against hantaviruses, alongside newer mRNA systems. Inactivated whole-virus vaccines against HTNV and SEOV are licensed in China and South Korea to prevent HFRS in endemic regions, but their protective efficacy remains uncertain [9, 37]. DNA vaccines encoding viral glycoproteins have undergone clinical trials with demonstration of immunogenicity but uncertain real-world efficacy.

Phase I clinical trials performed for Andes, Hantaan, and Puumala variants indicate that conventional vaccines induce neutralizing antibodies but still require several boosting [37]. To overcome this limitation, Meyer et al. recently evaluated lipid nanoparticle-formulated mRNA vaccines encoding the ANDV envelope glycoproteins Gn and Gc [38]. In the golden Syrian hamster model, this vaccine candidates demonstrated complete protection against lethal challenge and sterile immunity in the liver after only a single intramuscular dose, even at concentrations as low as 1 ${{{\rm{\mu }}}}{\mbox{g}}$. Dose-dependent IgG responses were detected as early as 14 days post-vaccination. These findings provide a strong scientific rationale for single-dose rapid-response deployment and potential post-exposure prophylaxis, offering a promising pathway toward clinical licensure and the containment of future outbreaks.

Furthermore, to facilitate future vaccine design, structural insights like high-resolution in situ structures of surface proteins are being deployed alongside hantavirus Gn nanoparticle structures to enhance target immunogenicity [37, 39].

## Synthesis: core theoretical disagreements and integrative framework

### Protection versus pathology in immune responses

The central paradox of hantavirus immunobiology is that the same immune mechanisms responsible for viral clearance are the primary drivers of tissue damage. CD8^+^ T cells and NK cells that eliminate infected endothelial cells simultaneously destroy vascular barrier integrity [3, 25]. The Treg deficit observed in patients suggests that the regulatory dimension of immunity, rather than the effector dimension per se, may be the critical variable distinguishing controlled resolution from immunopathological escalation [26, 27].

### Viral evasion as pathogenic amplifier

A counterintuitive but empirically supported proposition is that viral immune evasion mechanisms actually amplify disease severity by delaying immune engagement and permitting wider viral dissemination [21, 22, 40]. This creates a temporal mismatch: by the time effector responses engage, the infection is sufficiently widespread that clearance requires an immunopathologically destructive level of cytotoxic activity.

### Organ tropism determinants

The molecular basis for the HFRS/HCPS distinction—renal versus pulmonary target organ involvement—remains incompletely defined. While glycoprotein sequences encoded by the M segment are implicated [13], the downstream mechanisms through which differential receptor usage translates into organ-specific pathology involve complex interactions between viral tropism, local immune microenvironments, and endothelial bed heterogeneity that are not yet fully elucidated.

**Future Research Agenda**

1. **Mechanisms of Immune Calibration Failure:** Investigating the factors that distinguish mild or subclinical infections from severe immunopathology. Beyond specific HLA genotypes, future studies should focus on the kinetics of early Treg induction, the timing of the innate-to-adaptive transition, and how initial viral inoculum dose influences the disease trajectory. Longitudinal cohorts that capture the pre-symptomatic immune phase are critically needed.
2. **The Receptor-Tropism-Pathogenesis Axis:** Mapping how distinct receptor engagements (such as α_v_β_3_ integrin versus PCDH1) interact with structural variations in different endothelial beds. Applying single-cell transcriptomic profiling to infected endothelial tissue from various organs could help clarify the mechanisms behind organ-specific pathology.
3. **Optimizing Therapeutic Windows:** Managing the temporal divide between viral replication and immunopathology. Antivirals are most effective early in the lifecycle when diagnosis is difficult, whereas immunomodulators are needed later when viral clearance may be incomplete. Developing biomarker-guided protocols to identify the optimal transition point between antiviral and immunomodulatory therapies is a key priority.
4. **Characterizing Reservoir Host Tolerance:** Investigating the molecular mechanisms that allow natural rodent reservoirs to tolerate persistent infections. Understanding how these hosts maintain Treg activity and suppress inflammatory cytokines could help identify novel therapeutic targets for human disease.
5. **Reassortment Potential and Biosecurity Risk:** Assessing the likelihood of genetic reassortment among co-infecting lineages. Evaluating whether reassortment could combine the person-to-person transmissibility of ANDV with the higher mortality rates of other hantaviruses is a critical focus for global public health and biosecurity preparedness.
